# Na^+^/K^+^-ATPase α1 subunit, a novel therapeutic target for hepatocellular carcinoma

**DOI:** 10.18632/oncotarget.4726

**Published:** 2015-08-18

**Authors:** Liping Zhuang, Litao Xu, Peng Wang, Yan Jiang, Pan Yong, Chenyue Zhang, Haibin Zhang, Zhiqiang Meng, Peiying Yang

**Affiliations:** ^1^ Department of Integrative Medicine, Fudan University Shanghai Cancer Center, Department of Oncology, Shanghai Medical College, Fudan University, Collaborative Innovation Center for Cancer Medcine, Shanghai, China; ^2^ Eastern Hepatobilliary Surgery Hospital, Second Military Medical University, Shanghai, China; ^3^ Department of Palliative, Rehabilitation and Integrative Medicine, The University of Texas MD Anderson Cancer Center, Houston, TX, USA

**Keywords:** Na^+^/K^+^-ATPase, subunit, hepatocellular carcinoma, oxidative stress

## Abstract

We aimed to identify the expression patterns of Na^+^/K^+^-ATPase (NKA) α subunits in human hepatocellular carcinoma (HCC) samples and evaluate these subunits as potential targets for HCC treatment. The mRNA expression profiles of NKA α subunits in human HCC samples were analyzed. We found that the mRNA expression for NKA α1 subunit (ATP1A1) was higher than that for other NKA α subunits. Also, ATP1A1 gene expression was markedly higher in HCC samples than in adjacent nontumor tissue samples. Western blotting data suggested that 6 of 14 (43%) HCC samples had elevated ATP1A1 protein expression. Furthermore, knockdown of ATP1A1 expression in human HCC HepG2 and MHCC97H cells markedly reduced their proliferation *in vitro* and suppressed the tumorigenicity of MHCC97H cells *in vivo*. Downregulation of ATP1A1 expression resulted in cell-cycle arrest at G2/M phase and apoptosis in HepG2 cells as well as decreased migration in Hep3B cells. We further validated that ATP1A1 downregulation caused intracellular accumulation of reactive oxygen species. Pretreatment with *N*-acetyl cysteine blocked cell-growth inhibition induced by ATP1A1 downregulation. Collectively, these data suggested that targeting ATP1A1 is a novel approach to the treatment of HCC.

## INTRODUCTION

Hepatocellular carcinoma (HCC) is one of the most lethal cancers. Surgical resection at an early stage is the only curative treatment option, but more than 80% of HCC cases are diagnosed at an advanced stage with inoperable distant metastases [1]. Physicians have made advances in clinical management recently, and patients with unresectable HCC can benefit from multimodality treatment options, such as percutaneous ethanol injection, radiofrequency ablation, transarterial chemoembolization, high-intensity focused ultrasound, and magnetic resonance-guided laser thermal ablation [2–3]. However, the effectiveness of agents used in systematic treatment of HCC is relatively limited. For example, even though sorafenib is approved by the Food and Drug Administration as a standard of care in treatment of advanced HCC, it prolonged the median overall survival (OS) duration in HCC patients by only about 3 months, and long-term survival remains low [4]. Thus, searching for new therapeutic targets and pertinent novel compounds for HCC treatment remains highly important.

Na^+^/K^+^-ATPase (NKA) is the most prominent member of the P-type ATPase family, which comprises three types of polypeptide: the α and β subunits and the FXYD proteins [5]. Investigators have identified four α and four β subunits to date [6]. Also, researchers have extensively studied the transport function of NKA. Specifically, it pumps three Na^+^ ions out of and two K^+^ ions into a cell per molecule of hydrolyzed ATP, thereby creating an electrochemical gradient across the plasma membrane. In addition, NKA is a multifunctional protein that plays roles in cell junctions, adhesion, motility, and signal transduction [7–10]. NKA holds promise as a novel target in anticancer therapy given its aberrant expression and activity in various types of human cancer [11–13]. However, the dysregulation of NKA subunits varies among cancers; thus, its role in tumor development remains unclear. For instance, authors have reported overexpression of NKA α1 subunit (ATP1A1) in non-small-cell lung cancer [14], renal clear cell carcinoma [15], glioblastoma [16], and melanoma [17] cases but low expression in colorectal cancer cases [18]. We hypothesized that NKA α subunits were dysregulated in HCC and played roles in the biological capabilities of HCC cells. In the present study, we set forth to identify the expression patterns for NKA α subunits in HCC samples and evaluate their potential as novel targets for HCC treatment.

## RESULTS

### ATP1A1 expression is higher in HCC samples than in adjacent nontumor tissue samples

**Figure 1:**
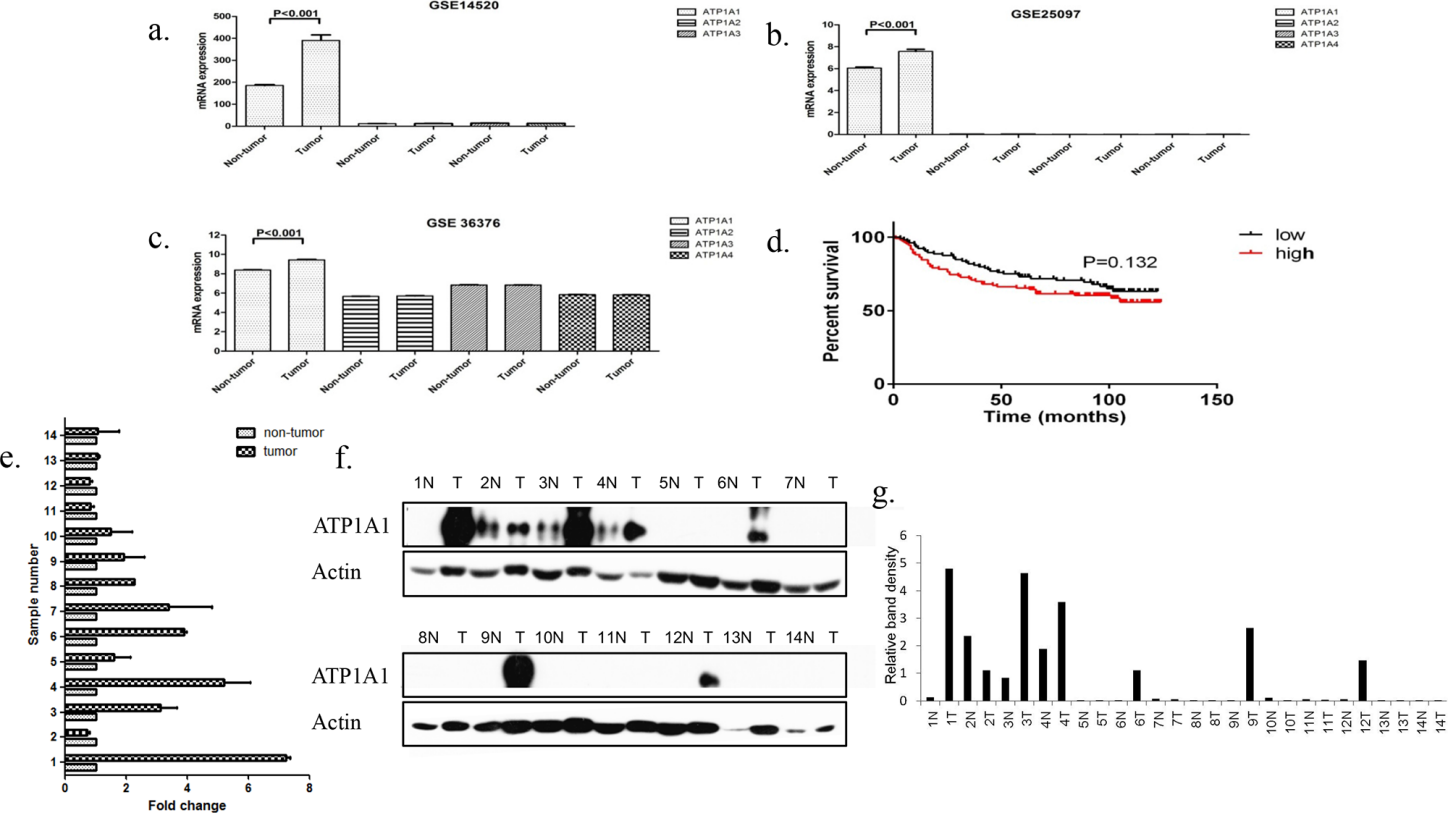
ATP1A1 is overexpressed in HCC samples **a–c.** The mRNA expression for ATP1A1 subunit was higher in HCC samples than in adjacent normal tissue samples in three different data sets. **d.** Kaplan-Meier curve of OS in a cohort of 240 HCC patients grouped by ATP1A1 mRNA expression (high versus low). The median ATP1A1 expression level was chosen as the cutoff point for separating the low and high ATP1A1-expressing cases (*n* = 120/group). Patients with high ATP1A1 expression tended to have poor survival durations, but the difference in OS was not significant (*P* = 0.132). **e.** ATP1A1 mRNA expression in 14 HCC samples compared with that in adjacent normal tissue samples. **f and g.** Qualitative and quantitative analysis of the ATP1A1 protein expression in 14 HCC samples compared with that in adjacent normal tissue samples. The relative band density was normalized according to actin.

To identify the expression patterns for NKA α subunits in HCC samples, we analyzed their mRNA expression in a large number of human HCC samples with data contained in the Gene Expression Omnibus, a public functional genomic data repository supporting Minimum Information about a Microarray Experiment-compliant data submissions [19]. Three expression data sets GSE 14520 [20], GSE 25097 [21], and GSE 36376 [22] were included in the analysis. Data from the three data sets are shown in Supplementary Table S1. The results demonstrated that expression of the ATP1A1 gene was significantly higher in HCC samples than in adjacent nontumor tissue samples in all three data sets from the Gene Expression Omnibus (Figures 1a–1c). We then analyzed the relationship between ATP1A1 mRNA expression and patient survival using the GSE 36376 data set. We grouped the patients into low and high ATP1A1 expression groups using the median expression level in all cases as the cutoff point. Kaplan-Meier analysis demonstrated that median survival of patients with high ATP1A1 was 86.7 months while the patients with low ATP1A1 was 96.2 months (*P* = 0.132) (Figure 1d). Overall, ATP1A1 mRNA expression was upregulated in HCC samples.

We further examined the ATP1A1 mRNA and protein expression in 14 pairs of HCC samples obtained from the tumor bank at Eastern Hepatobilliary Surgery Hospital (Shanghai, China). We found that nine HCC samples (64.3%) had higher ATP1A1 mRNA expression than that in adjacent nontumor tissue samples (Figure 1e). In addition, six HCC samples (43%) had higher ATP1A1 protein expression than that in adjacent nontumor tissue samples (Figures 1f and 1g). According to the results of western-blot, patients were divided into ATP1A1-high and ATP1A1-low group. Survival analysis showed that The DFS in patients with ATP1A1-low and ATP1A1-high group were 936.7 ± 865.5 days and 514 ± 412.4 days, respectively (*P* = 0.3395, Supplementary Figure S1).

### Downregulation of ATP1A1 expression in human HCC cells results in proliferation arrest

**Figure 2:**
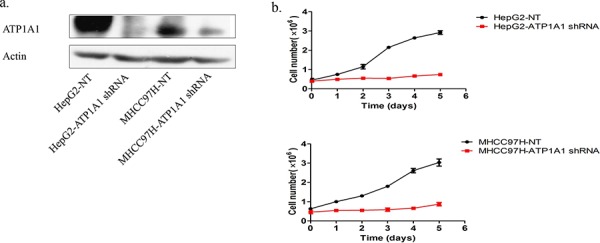
Downregulation of ATP1A1 expression in human HCC cells results in proliferation arrest **a.** The ATP1A1 protein expression in HepG2 and MHCC97H HCC cell lines transfected with ATP1A1-shRNA. **b.** Growth of HepG2 (top) and MHCC97H (bottom) HCC cells transfected with nontargeted shRNA (NT) and ATP1A1-shRNA. The cells were counted every 24 h.

**Figure 3:**
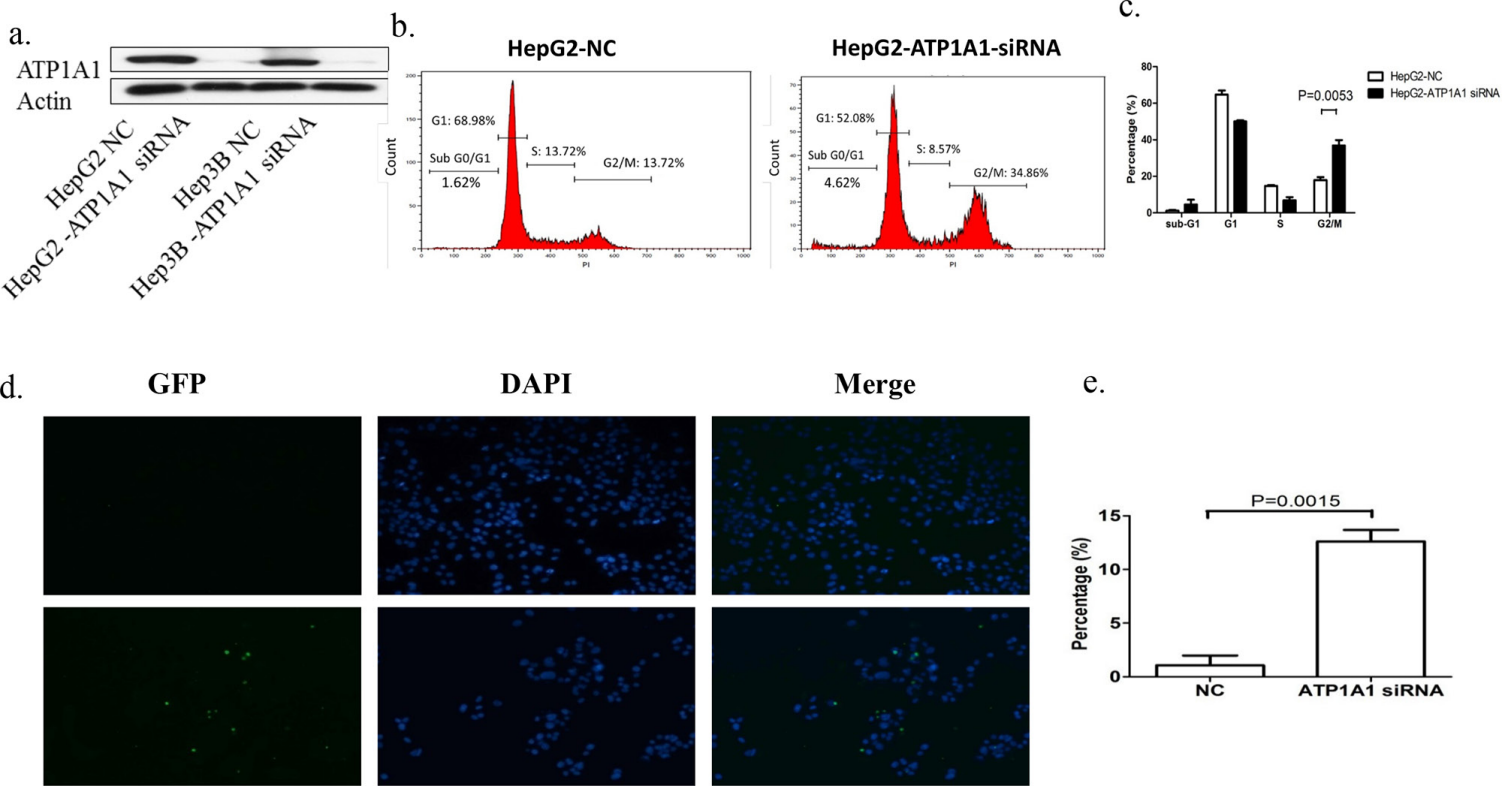
Downregulation of ATP1A1 expression in human HCC cells results in cell-cycle arrest at G2/M phase and apoptosis **a.** ATP1A1 protein expression in HepG2 and Hep3B HCC cells transfected with ATP1A1-siRNA. **b.** Alteration of the cell-cycle phases in HepG2 cells by knockdown of ATP1A1 expression. Left, cell-cycle distribution in HepG2 cells transfected with scrambled siRNA (NC). Right, cell-cycle distribution in HepG2 cells transfected with ATP1A1-siRNA. **c.** Histogram showing the percentage of HepG2 cells in the different cell-cycle phases (three independent experiments). **d.** Apoptotic cell death resulting from downregulation of ATP1A1 expression in human HCC cells. Top row, death of HepG2 cells transfected with scrambled siRNA (NC). Bottom row, death of HepG2 cells transfected with ATP1A1-siRNA. GFP, green fluorescent protein; DAPI, 4′,6-diamidino-2-phenylindole. **e.** Histogram showing the percentage of apoptotic HepG2 cells (independent experiments performed in triplicate).

We constructed three short hairpin RNA (shRNA) pools specifically targeting ATP1A1. Up to 80% of HepG2 and MHCC97H cells transfected with an ATP1A1-shRNA vector exhibited reduced ATP1A1 expression (Figure 2a). Also, proliferation of these cells was notably lower than that of cells transfected with nontargeted shRNA (Figure 2b). We also studied the effect of reduced ATP1A1 expression induced by transfection with small interfering RNA (siRNA) on cell-cycle distribution using flow cytometric analysis of cellular DNA content. As shown in Figure 3a, HepG2 cells transfected with ATP1A1-siRNA had remarkably reduced ATP1A1 gene expression. ATP1A1 downregulation in HCC cells resulted in arrest of cells at the G2/M phase of the cell cycle. The mean (± standard deviation [SD]) G2/M cell-cycle distributions in HepG2 cells transfected with scrambled siRNA and ATP1A1-siRNA were 18.0% ± 2.8% and 36.9% ± 5.2%, respectively (*P* < 0.01). This increase in the arrest of cells at G2/M phase was accompanied by a concomitant decrease in the arrest of cells at the G1 and S phases. Taken together, these data suggested that induction of cell-cycle arrest at G2/M phase in HCC cells is responsible for the cell-growth inhibition induced by downregulation of ATP1A1 expression (Figures 3b and 3c).

### Downregulation of ATP1A1 expression induces moderate apoptotic cell death

In our cell-cycle analysis, we also found an increase of HepG2 cells at sub-G1 phase after knockdown of ATP1A1 expression. We next examined HepG2 cell apoptosis using a terminal deoxynucleotidyl transferase dUTP nick end labeling (TUNEL) assay. The mean (± SD) percentage of apoptotic ATP1A1-siRNA–transfected cells (12.6% ± 1.5%) was significantly higher than that of scrambled siRNA-transfected cells (1.06% ± 1.3%) (Figures 3d and 3e). Pro-apoptotic effect was also noted in Hep3B cells after ATP1A1 knockdown (Supplementary Figure S2).

### Downregulation of ATP1A1 expression impairs the migration of HCC cells

**Figure 4:**
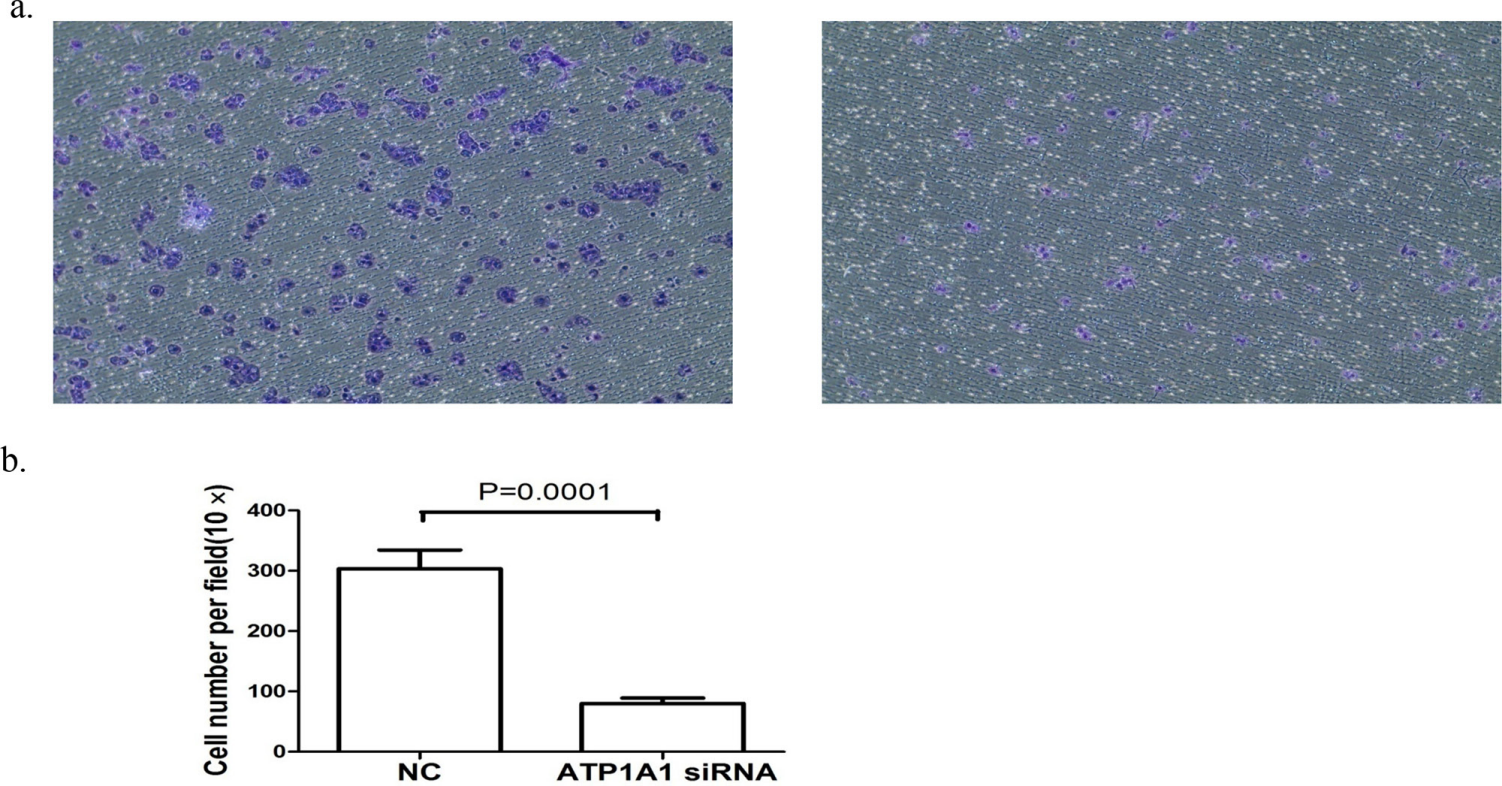
Downregulation of ATP1A1 expression in human HCC cells results in reduced cell migration **a.** Migration of Hep3B cells transfected with (left) control siRNA (NC) and (right) ATP1A1-siRNA as analyzed using a Transwell assay. **b.** Histogram showing the numbers of migrating cells stained with crystal violet (independent experiments performed in triplicate).

NKA plays a critical role in the formation and maintenance of tight junction structures and permeability in epithelial cells [23–25]. Thus, we examined the migration of Hep3B HCC cells after knockdown of ATP1A1 expression in them. The number of migrating ATP1A1-knockdown cells was substantially lower than that of control siRNA-transfected cells (Figure 4a). The reduction in migration induced by ATP1A1 knockdown was statistically significant (*P* = 0.0001) (Figure 4b).

### Downregulation of ATP1A1 expression affects the tumorigenicity of MHCC97H cells *in vivo*

**Figure 5:**
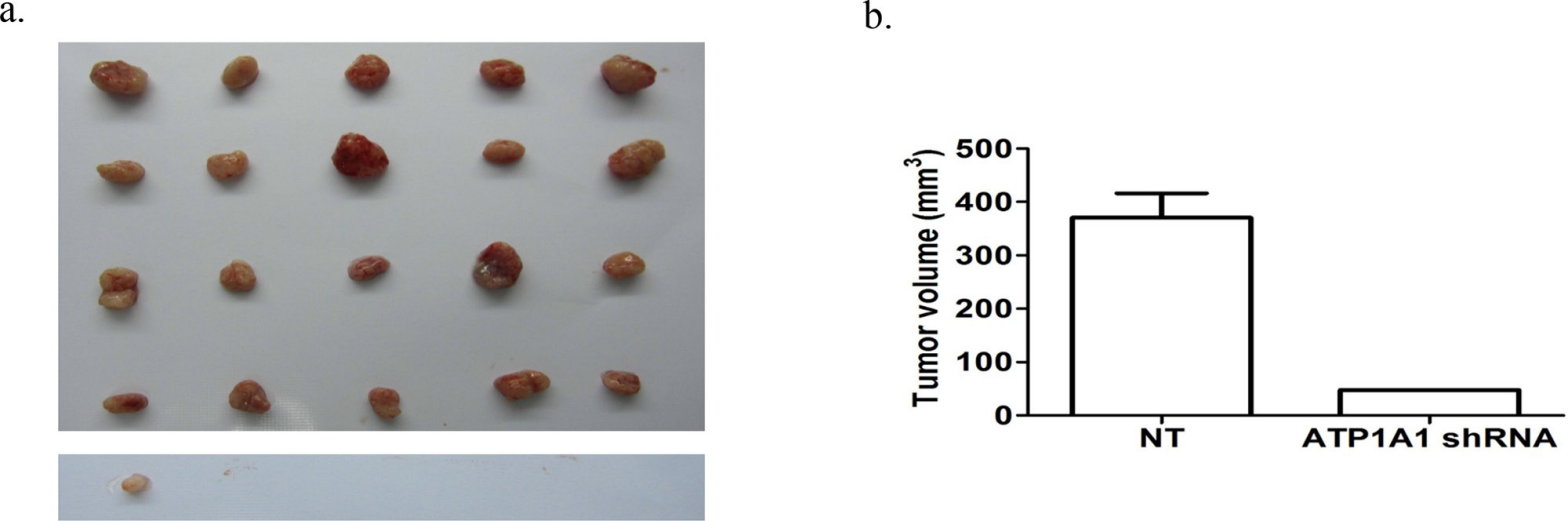
Downregulation of ATP1A1 expression affects the tumorigenicity of MHCC97H cells *in vivo* **a.** Tumors generated by MHCC97H cells transfected with (top) nontargeted shRNA and (bottom) ATP1A1-shRNA and implanted subcutaneously into nude mice. Tumor growth was observed after 40 days. **b.** Histogram showing the mean tumor volumes in the two groups of mice.

We subcutaneously implanted MHCC97H cells transfected with nontargeted shRNA and ATP1A1-shRNA in nude mice. In the nontargeted shRNA-transfected MHCC97H group, the tumorigenicity rate was 100%, as subcutaneous solid tumors developed in all of the animals tested in this study. In contrast, we found only one tumor in the ATP1A1-shRNA-transfected MHCC97H group 40 days after implantation (Figure 5a). The mean volume of the xenograft tumors generated by ATP1A1-shRNA–transfected MHCC97H cells (47.9 mm^3^) was notably smaller than that of the tumors generated by nontargeted shRNA-transfected MHCC97H cells (370.0 mm^3^) (Figure 5b).

### Downregulation of ATP1A1 expression causes HCC cells to undergo oxidative stress

To understand the mechanisms associated with ATP1A1 knockdown-elicited reduction of the tumorigenicity of HCC cells, we used a HumanHT-12 v4 array (Illumina, San Diego, CA, USA) to identify these mechanisms in HepG2 cells. We found that 5226 of a total of 34,694 genes differed significantly between the two groups of cells transfected with scramble siRNA and ATP1A1-siRNA (*P* < 0.05). Among these 5226 genes, expression of 4164 genes was downregulated, whereas that of 1062 genes was upregulated. The top 10 down-regulated and up-regulated genes after downregulation of ATP1A1 expression are shown in Table 1. We then used the genes with significantly different expression to enrich the pathways using Reactome FI Cytoscape Plugin 4. The results demonstrated that knockdown of ATP1A1 expression impaired the genes that correlated with the cell cycle and metabolism (Table 2). The pathway enrichment indicated that knocking down ATP1A1 expression may increase oxidative stress as evidenced by alteration of the expression of genes associated with oxidation, such as GSTA1, GSTA4, ACOX2, ALDH6A1, UCP2 and LOX, which were validated by q-PCR (Supplementary Figure S3).

**Table 1:** Top down-regulated and up-regulated genes in HepG2 cells after ATP1A1 downregulation by PCR validation

	Gene symbol	Gene description	RQ
**Downregulated**	PFKFB4	6-phosphofructo-2-kinase/fructose-2,6-biphosphatase 4	0.1
	ATP1A1	ATPase, Na+/K+ transporting, alpha 1 polypeptide	0.11
	HP	haptoglobin	0.18
	STMN3	stathmin-like 3	0.26
	LOX	lysyl oxidase	0.28
	LIME1	Lck interacting transmembrane adaptor 1	0.41
	KANK4	KN motif and ankyrin repeat domains 4	0.54
	VGF	VGF nerve growth factor inducible	0.64
	NREP	neuronal regeneration related protein	0.64
**Upregulated**	RAMP1	receptor (G protein-coupled) activity modifying protein 1	54.2
	GDF15	growth differentiation factor 15	25.6
	TMEM27	transmembrane protein 27	17.8
	FAM46C	family with sequence similarity 46, member C	10.7
	SLC5A3	solute carrier family 5, member 3	7.5
	GSTA1	glutathione S-transferase alpha 1	5.6
	AKR1B1	aldo-keto reductase family 1, member B1	4.8
	SERPINE2	serpin peptidase inhibitor, clade E, member 2	4.7
	C9ORF152	chromosome 9 open reading frame 152	4.5
	CA2	carbonic anhydrase II	4.2

**Table 2:** Reactome Pathways involved in the downregulation of ATP1A1 expression in HCC cells

*Reactome pathway*	*Branch*	*FDR*
Cell cycle	Cell-cycle checkpoints (G2/M checkpoints)	<1.111e-04
	Cell cycle, mitotic	<3.333e-04
	Chromosome maintenance	<5.882e-05
Cellular responses to stress	Cellular senescence	8.990e-05
DNA repair	Nucleotide excision repair	2.632e-04
DNA replication	M/G1 transition	<2.632e-05
	Synthesis of DNA	<5.000e-05
Metabolism	Metabolism of lipids and lipoproteins	<3.704e-05
	The citric acid cycle and respiratory electron transport	<2.564e-05
	Metabolism of nucleotides	<2.439e-05
	Metabolism of vitamins and co-factors	<1.316e-05
	Metabolism of amino acids and derivatives	<9.091e-05
	Biological oxidations	<2.381e-05

Abbreviation: FDR, false discovery rate.

**Figure 6:**
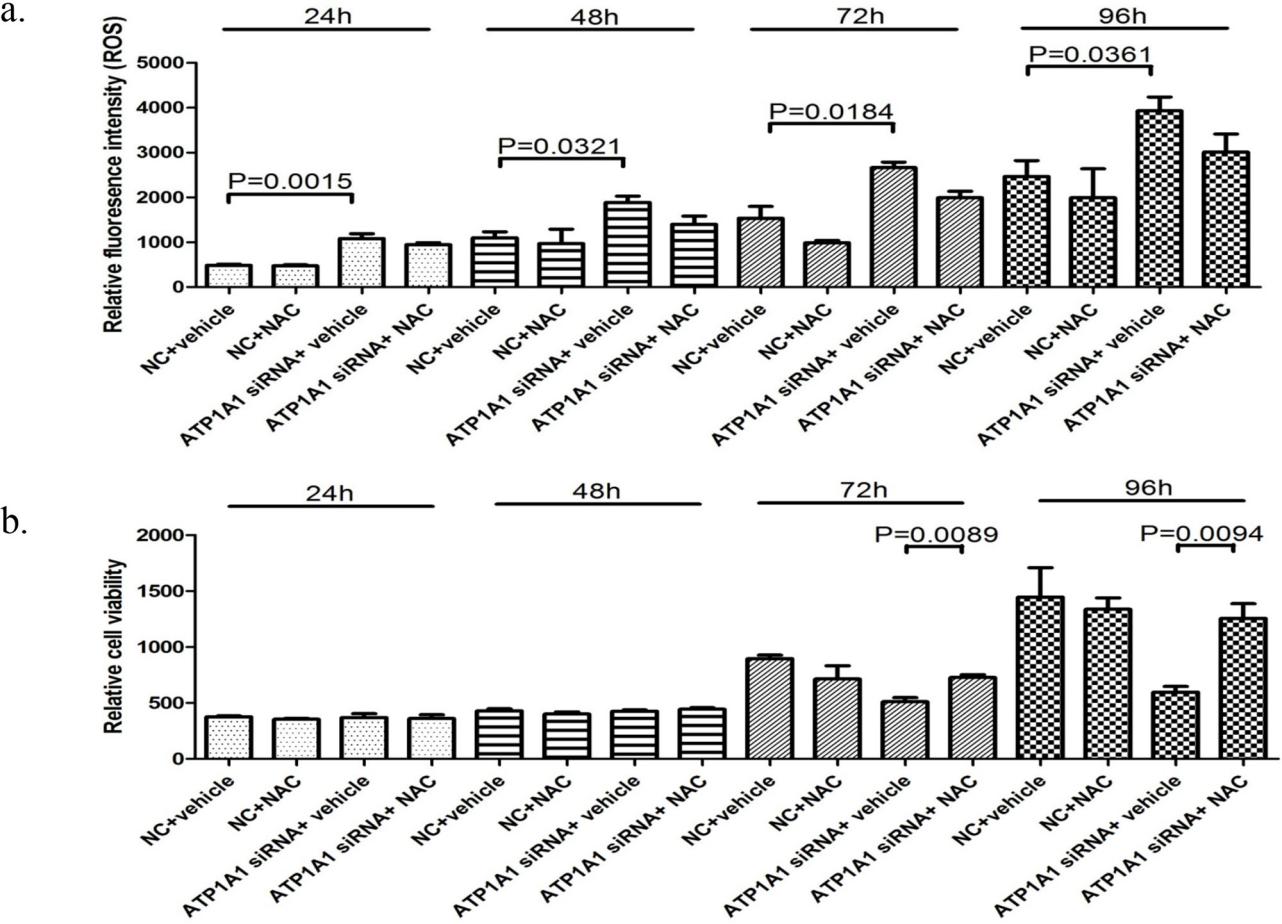
Downregulation of ATP1A1 expression causes HCC cells to undergo oxidative stress **a.** ATP1A1 downregulation-mediated formation of ROS. Knockdown of ATP1A1 expression in HepG2 cells resulted in increased formation of ROS as measured according to cellular fluorescence derived from pretreatment of cells with H_2_DCF-DA dye. Also shown is inhibition of ROS formation by the use of the antioxidant glutathione precursor NAC (10 mM). The data shown are presented as the mean ± SD (*n* = 3). **b.** ATP1A1 downregulation-mediated inhibition of cell growth was partially inhibited by co-incubation with 10 mM NAC. NC, scrambled siRNA.

We then determined whether ATP1A1 downregulation affects the formation of reactive oxygen species (ROS) via measurement of the formation of fluorescent 2′, 1′-dichloro fluorescein diacetate (H_2_DCF-DA) as a result of an oxidative reaction with intracellular free radicals. As shown in Figure 6a, ATP1A1 downregulation-mediated ROS formation in HepG2 cells increased two-fold after transfection of siRNA for 24 hours. The increased ROS formation in ATP1A1-knockdown HepG2 cells continued for up to 96 hours. Intriguingly, intracellular formation of both endogenous and ATP1A1 downregulation-mediated ROS was inhibited by exposure of the cells to *N*-acetyl cysteine (NAC), an antioxidant and precursor of glutathione. In addition, we examined the effect of NAC exposure on ATP1A1 downregulation-mediated inhibition of cell growth in HepG2 cells (Figure 6b). NAC provided near complete protection against ATP1A1 downregulation-induced inhibition of cell growth at both 72 and 96 h.

## DISCUSSION

The results of the present study strongly suggest that ATP1A1 expression was higher in a large proportion of clinical HCC samples than nontumor liver tissue samples. Knockdown of ATP1A1 expression remarkably reduced the proliferation and migration and increased apoptosis in human HCC cells *in vitro* and impaired their tumorigenicity *in vivo*, further indicating that ATP1A1 plays an important role in HCC development and is a potential target for HCC treatment.

As an ion transport pump, NKA not only is critical for maintenance of the homeostasis of Na^+^ and K^+^ but also plays a critical role in cellular function and signaling [26–27]. Thus, downregulation of ATP1A1 expression in cancer cells is expected to affect several processes of cellular function. Using a genome-wide microarray, we found that genes associated with the cell cycle and metabolism were affected by ATP1A1 downregulation in HCC cells. We further found that cells underwent oxidative stress following ATP1A1 downregulation evidenced by increased intracellular accumulation of ROS in ATP1A1 knockdown HCC” cells. Pretreatment with NAC blocked cell-growth inhibition induced by ATP1A1 downregulation, implying that ROS generation is essential for ATP1A1 knockdown-mediated proliferation arrest. Excessive ROS formation may cause oxidative injury of DNA [28], which can induce apoptosis and cell-cycle arrest via checkpoint activation [29–30].

Cardiac glycosides, a class of steroid-like compounds that include well-known drugs such as digoxin and digitoxin, are inhibitors of NKA activity. Over the past 3 decades, researchers have suggested that cardiac glycosides inhibit cell proliferation and exert valuable cytotoxic activity against different cancer cell lines [31–33]. Cardiac glycosides are now being studied in several clinical trials to determine their possible roles in cancer treatment [34]. Different cardiac glycosides exhibit varied levels of binding affinity to each α subunit of NKA. Hauck *et al*. [35] reported that digoxin (α2 = α3 > α1) and β-acetyldigoxin (α1 > α3) had distinct isoform-specific affinities but that ouabain, digitoxin, and methyldigoxin did not have any differences in isoform-specific affinities. Because the α subunit expression patterns in HCC samples demonstrated increased α1 expression, the cardiac glycosides used for HCC treatment should be those with greater affinity for α1than for other α subunits.

How inhibition of α1 expression increases oxidative stress as evidenced by increased formation of ROS in HepG2 cells remains unclear. We previously reported that treatment with the cardiac glycoside oleandrin inhibits the proliferation of BRO human melanoma cells by increasing ROS generation [36]. Excessive ROS production results in DNA damage and activates cell-cycle checkpoints, leading to cell-cycle arrest and prevention of the replication of damaged and defective DNA [37]. Also, activation of p53 is a frequent response to oxidative stress and is critical for cell-cycle-checkpoint regulation [38]. Our preliminary data demonstrated that compared to HepG2 cells transfected with scrambled siRNA, HepG2 cells transfected with ATP1A1-siRNA had higher expression of p53 and phosphorylation of p53 on serine 15. Pretreatment with NAC could partially reduce the activation of p53 in HepG2 cells transfected with ATP1A1siRNA (Supplementary Figure S4). Thus, increased ROS production resulted in p53 activation could be one of the mechanism for the proliferation arrest upon ATP1A1 silencing. Studies demonstrated that cells with expressing a dominant-negative form of Sir2 potentiate p53 activity in response to oxidative stress [39–40]. Additionally, p53 can increase ROS levels via its downstream effector p66^SHC^ [41–42]. However, there must be other potential mechanisms involved in the effect of ATP1A1 silencing on cell proliferation in p53 mutant or p53 null cells, which needs further investigation.

The limitations of our study: Firstly, limited tissue samples were available to determine the protein expression of ATP1A1. For relationship between ATP1A1 protein expression and patient survival, the sample size was too small to reach the statistical significance. Second, the underlying mechanisms of impacts of ATP1A1 knockdown on liver cancer cells need further explorations.

In conclusion, we demonstrated for the first time overexpression of ATP1A1 in a large number of clinical HCC samples. ATP1A1 downregulation remarkably reduced HCC cell proliferation *in vitro* and impaired cell tumorigenesis *in vivo*. Thus, ATP1A1 could be considered a potential therapeutic target for HCC.

## MATERIALS AND METHODS

### Cell culture and cell proliferation assay

HepG2 and Hep3B cells were obtained from the American Type Culture Collection (Manassas, VA, USA). MHCC97H cells were obtained from Dr. Z. Y. Tang at Zhongshan Hospital, Fudan University (Shanghai, People’s Republic of China) [43]. These cells were maintained in Dulbecco’s modified Eagle’s medium (DMEM) supplemented with 10% heat-inactivated bovine serum and kept at 37°C in an atmosphere of 95% air and 5% CO_2_. To assess cell proliferation, 4 × 10^5^ cells were seeded in six-well plates. The cell number was determined every other day using an automated cell counter.

### Cell-cycle analysis

Cell-cycle analysis of synchronized cells was monitored by examining the cells’ DNA profiles after staining with propidium iodide. Cells were harvested, washed with 1× phosphate-buffered saline (PBS), fixed in 70% cold ethanol at 4°C for 10 min, hydrolyzed with 5 mg/ml ribonuclease A for 20 min, and stained with 50 μg/ml propidium iodide. The DNA content in the cells was determined using a flow cytometer (FACSVANTAGE; BD, San Jose, CA, USA) equipped with a 488-nm argon laser, and the cell populations in the different cell-cycle phases were quantified using the ModFit software program (Verity Software House, USA). This experiment was performed in triplicate.

### TUNEL assay

Cells were fixed in 4% paraformaldehyde for 1 h at room temperature. Cells were then rinsed with PBS and incubated in 0.1% Triton X-100 for 2 min on ice. They were then washed twice with PBS and incubated in a TUNEL reaction mixture for 1 h at 37°C in a humidified atmosphere in the dark. The cells that underwent apoptosis were examined under a fluorescence microscope at a magnification of 20× (EVOS Cell Imaging Systems; AMG, Mill Creek, WA, USA).

### Cell migration assay

A cell migration assay was carried out using Transwell filter chambers (BD Biosciences, San Jose, CA, USA). Briefly, 5 × 10^4^ Hep3B cells transfected with control siRNA or ATP1A1 siRNA were seeded into the top inserts of the chambers. DMEM containing 10% FBS was added to the receiver wells to trigger cell migration. After 6 h of incubation, a cotton swab moistened with medium was inserted into the chamber to get rid of the cells on the upper surface of the membrane. The cells were fixed using 100% ethanol and stained with 0.1% crystal violet. The migrated cells were counted in five selected fields under a microscope at a 20x magnification.

### Molecular cloning and lentivirus production

DNA oligonucleotides encoding ATP1A1-shRNA were inserted into the *Age*I and *Eco*RI restriction sites of the pLKO.1 plasmid. Three pairs of DNA sequences encoding ATP1A1-shRNA were used: pair 1, CCGGCCAGTTGTCTATTCATAAGAAGGATCCT TCTT ATGAATAGACAACTGGTTTTTG; pair 2, CCGGCCTGC TGACCTCAGAATCA TAGGATCCTATGATTCTGAGGT CAGCAGGTTTTTG; and pair 3, CCGGCGGCAGTG ATC TAAAGGACATGGATCCATGTCCTTTAGATC ACTGCC GTTTTTG. The constructed pLKO.1 plasmid was then introduced into 293FT cells together with the packaging plasmid psPAX2 (Addgene, Cambridge, MA, USA) and the envelope plasmid pMD2.G (Addgene). After 48 h of incubation, the medium containing the lentiviruses was collected and passed through a 0.45-mm filter.

### Transfection of ATP1A1 siRNA

HCC cells were plated in 6-well and 96-well plates and allowed to attach overnight. Transient transfection of scramble siRNA and ATP1A1 siRNA molecules was carried out using Lipofectamine RNAiMAX Transfection Reagent (Invitrogen) following the instructions of the manufacturer.

### HumanHT-12 v4 array

HepG2 cells (5 × 10^5^) were seeded in a six-well plate. After attachment, cells were transfected with scrambled siRNA or ATP1A1-siRNA for 48 h. Total RNA was then isolated from these cells using TRIzol reagent (Invitrogen, Carlsbad, CA, USA). The array analysis was performed in triplicate by Sequencing and Microarray Facility at The University of Texas MD Anderson Cancer Center (Houston, TX, USA).

### Western blots

Western blot analysis was performed according to standard procedures using antibodies against ATP1A1 (EMD Millipore, Billerica, MA, USA) and actin (Cell Signaling Technology, Danvers, MA, USA).

### ROS generation

Generation of intracellular ROS in HCC cells was measured using H_2_DCF-DA. This nonpolar compound is taken up by cells and converted via oxidation to the nonfluorescent derivative DCF by cellular esterases. HepG2 cells were seeded in 96-well plates and transfected with siRNA. Cells were then washed twice with phenol red-free DMEM prior to the addition of H_2_DCF-DA to cells for a final concentration of 10 μM. The fluorescence intensity was then read using an FLx800 fluorescence microplate reader (BioTek, Winooski, VT, USA) with excitation and emission wavelengths of 485 nm and 530 nm, respectively, 24, 48, 72, and 96 h after siRNA transfection. The fluorescence intensity was normalized according to cell number. To determine the inhibitory effect of an antioxidant on ATP1A1 downregulation-mediated formation of ROS, cells were incubated with 10 mM NAC at 37°C for 30 min prior to the transfection of siRNA.

### Tumor generation assay

Four-week-old male BALB/c-nu/nu nude mice were purchased from the Shanghai Institute of Materia Medica, Chinese Academy of Sciences (Shanghai, People’s Republic of China) and housed in laminar flow cabinets under specific pathogen-free conditions with food and water provided *ad libitum*. All experiments involving mice were performed in accordance with the National Institutes of Health guidelines for the care and use of laboratory animals. The study protocol was approved by the Committee on the Use of Live Animals in Teaching and Research at Fudan University. MHCC97H cells transfected with nontargeted shRNA or ATP1A1-shRNA (4 × 10^6^ cells in a 200-μl volume) were injected into the right and left axillae of individual mice. Tumor growth was then examined for the following 40 days. The length and width of the tumors (in millimeters) were measured weekly using calipers. The tumor volume was calculated using the formula (a × b^2^) × 0.5, in which a and b are the long and short dimensions, respectively. Both groups contained 10 mice.

### Clinical samples

Human HCC and adjacent normal liver tissue samples were collected at Eastern Hepatobiliary Surgery Hospital. All of the samples were collected after patients gave their informed consent to participate, and all of the experiments were approved by the institution’s Internal Review and Ethics boards.

### Data analysis

Data were expressed as the mean ± SD. The Kolmogorov-Smirnov test was used to determine the normality of quantitative data. To analyze differences between the two groups, Student *t*-test analysis of mean numerical data and Mann-Whitney *U*-test analysis of nonnumerical data were performed. The Kaplan-Meier method was used to compare the OS durations in the two groups of patients. Data were analyzed using the SPSS software program (version 20; IBM Corporation, Armonk, NY, USA). *P* levels less than 0.05 were considered statistically significant.

## SUPPLEMENTARY FIGURES AND TABLE


