# Supplementary material for: Na+/K+-ATPase α1 subunit, a novel therapeutic target for hepatocellular carcinoma
Source: Oncotarget. 2015 Aug 18;6(29):28183–93. doi: 10.18632/oncotarget.4726 (PMC4695053; doi:10.18632/oncotarget.4726)
Supplement: Supplementary file 1 [file oncotarget-06-28183-s001.pdf]

SUPPLEMENTARY FIGURES AND TABLE

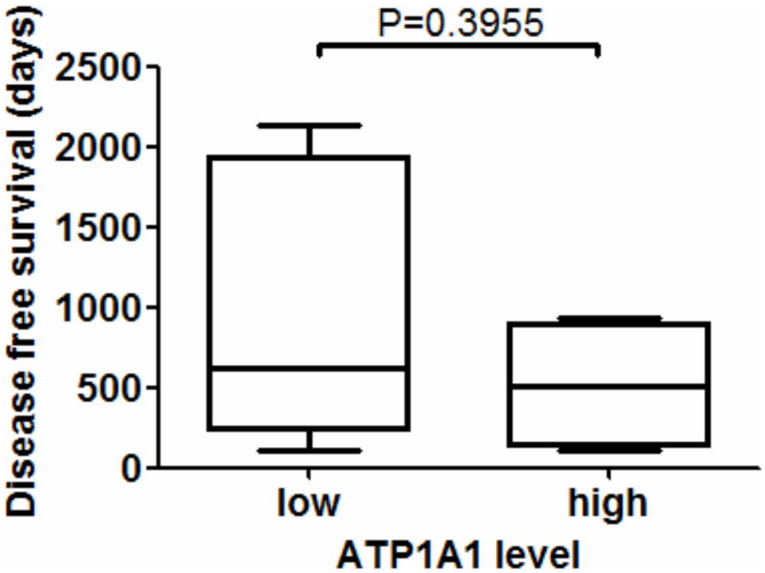

Supplementary Figure S1: According to the results of western-blot, patients were divided into ATP1A1-high and ATP1A1-low group. The disease free survival (DFS) in ATP1A1-low and ATP1A1-high group were 936.7 ± 865.5 days and 514 ± 412.4 days, respectively ($P = 0.3395$).

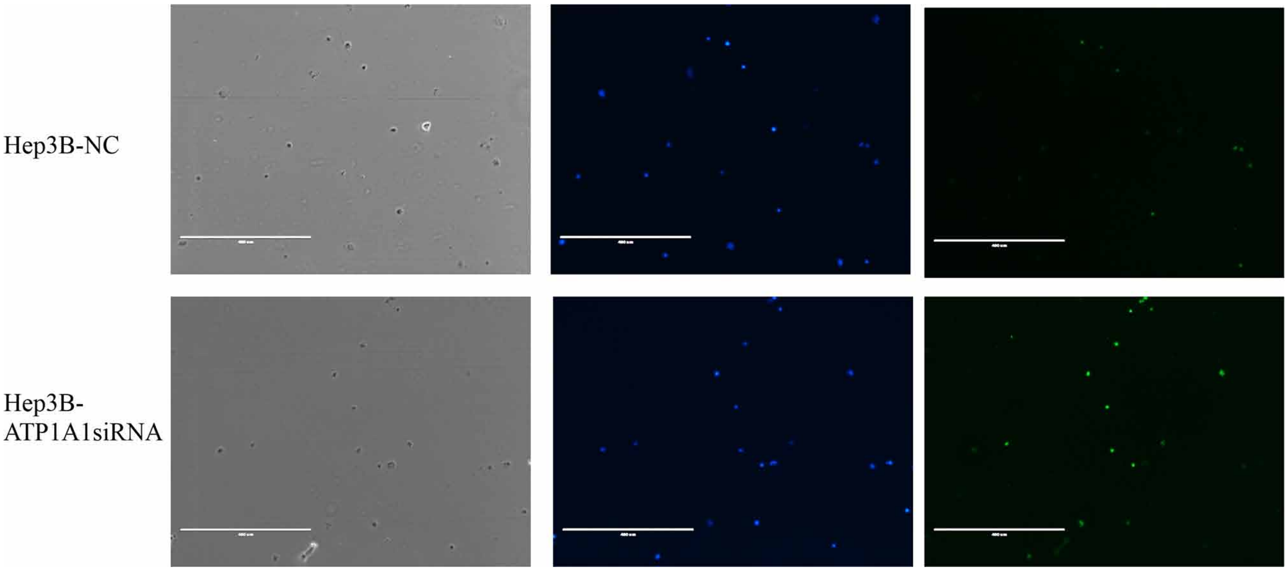

Supplementary Figure S2: TUNEL assay in Hep3B cells after ATP1A1 knockdown. Top row, death of Hep3B cells transfected with scrambled siRNA (NC). Bottom row, death of Hep3B cells transfected with ATP1A1-siRNA.

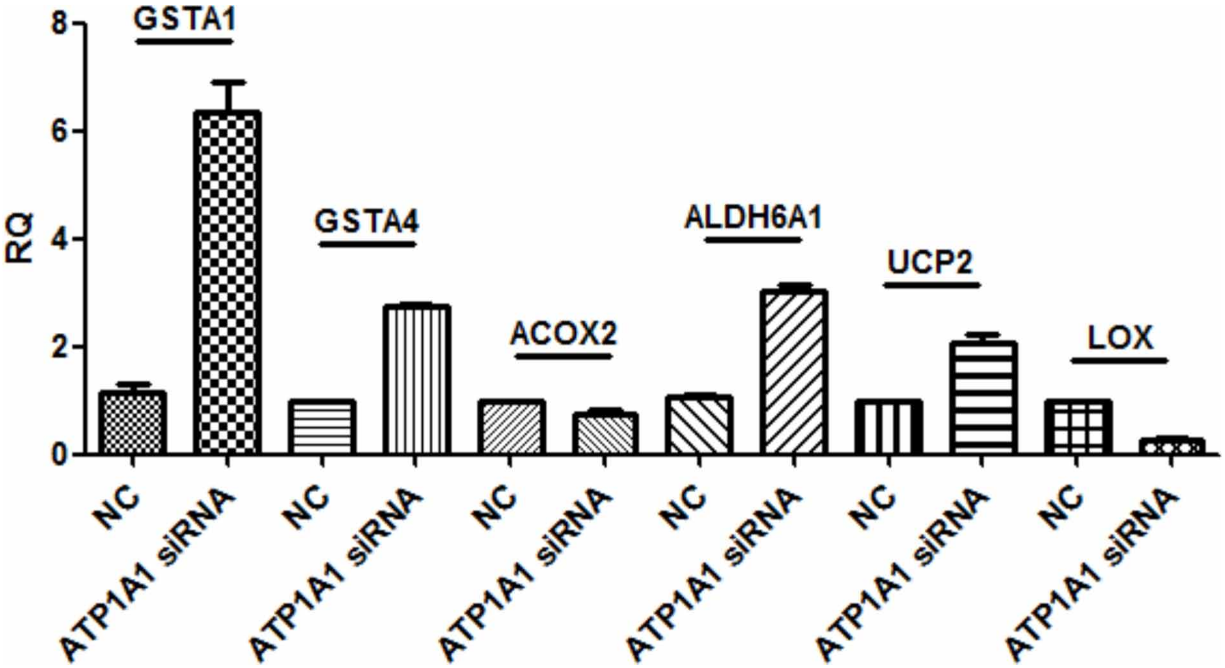

Supplementary Figure S3: The expression of genes associated with oxidation, such as GSTA1, GSTA4, ACOX2, ALDH6A1, UCP2 and LOX, which were validated by q-PCR.

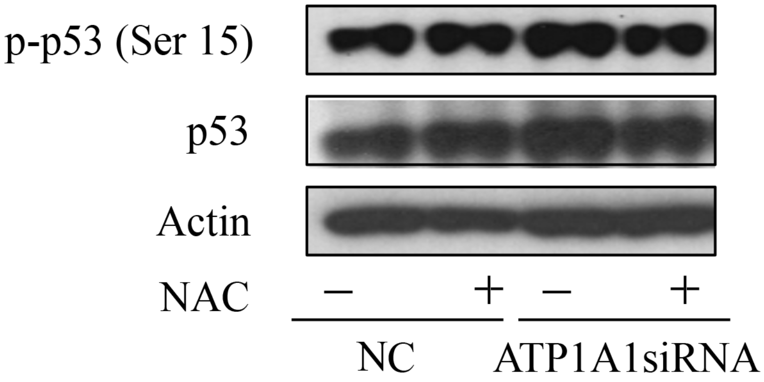

Supplementary Figure S4: ATP1A1 downregulation in HepG2 cells increases the expression of p53 and phosphorylation of p53 on serine 15. Pretreatment with NAC could partially reduce the activation of p53 in HepG2 cells transfected with ATP1A1siRNA. NC, scrambled siRNA.

Supplementary Table S1: Characteristics of the three datasets included in the analysis of mRNA level of α subunits in HCC samples

Dataset	*Country*	*Platform*	*Number of Samples*	*value*
GSE 14520	USA	Affymetrix Human Genome U133A 2.0 Array	434	log2 of signal intensity
GSE 25097	USA	Rosetta/Merck Human RSTA Affymetrix 1.0 microarray	511	normalized intensity
GSE 36376	Korea	Illumina HumanHT-12 V4.0 expression beadchip	433	quartile normalized
